# The effects of preosteoblast‐derived exosomes on macrophages and bone in mice

**DOI:** 10.1111/jcmm.18029

**Published:** 2023-11-06

**Authors:** Sema S. Hakki, Lena Batoon, Amy J. Koh, Rahasudha Kannan, Veronica Mendoza‐Reinoso, John Rubin, Laurie K. Mccauley, Hernan Roca

**Affiliations:** ^1^ School of Dentistry, Periodontics and Oral Medicine University of Michigan Ann Arbor Michigan USA; ^2^ Department of Periodontology, Faculty of Dentistry Selcuk University Konya Turkey; ^3^ Department of Biomedical Engineering University of Michigan Ann Arbor Michigan USA; ^4^ Department of Pathology, Medical School University of Michigan Ann Arbor Michigan USA

**Keywords:** bone, exosomes, extracellular vesicles, macrophages, osteoblasts, preosteoblast

## Abstract

The effect of preosteoblast‐derived exosomes on bone marrow macrophages (BMMΦ) and calvarial osteoblasts (cOB) was evaluated in vitro*,* and bone formation studies were performed in vivo in mice. Preosteoblastic MC3T3‐E1 clone 4 (MC4) cell‐derived exosomes (MC4exo) were characterized with particle tracking, transmission electron microscopy and western blot analysis to validate size, number, shape and phenotypic exosome markers. Exosomes pre‐labelled with PKH67 were incubated with BMMΦ and phagocytosis of exosomes was confirmed. To examine the effect of MC4exo on macrophage polarization, BMMΦ were treated with MC4exo and the expression of pro‐ and anti‐inflammatory cytokines was determined by qPCR. MC4exo treatment upregulated mRNA expression of *Cd86*, *Il1β*, *Ccl2*, *Rankl* and *Nos*, and downregulated *Cd206*, *Il10* and *Tnfα*, suggesting a shift towards pro‐inflammatory ‘M1‐like’ macrophage polarization. Combination of RANKL and MC4exo increased osteoclast differentiation of BMMΦ in comparison to RANKL alone as analysed by TRAP staining. MC4exo treatment showed no significant effect on calvarial osteoblast mineralization. For in vivo studies, intratibial inoculation of MC4exo (2 × 10^9^ particles in PBS, *n* = 12) and vehicle control (PBS only, *n* = 12) was performed in C57Bl/6 mice (8 weeks, male). Micro‐CT analyses of the trabecular and cortical bone compartments were assessed at 4 weeks post‐injection. Tibial sections were stained for TRAP activity to determine osteoclast presence and immunofluorescence staining was performed to detect osteocalcin (Ocn), osterix (Osx) and F4/80 expression. Intratibial inoculation of MC4exo increased the diaphyseal bone mineral density and trabecular bone volume fraction due to increased trabecular number. This increase in bone was accompanied by a reduction in bone marrow macrophages and osteoclasts at the experimental endpoint. Together, these findings suggest that preosteoblast‐derived exosomes enhanced bone formation by influencing macrophage responses.

## 1 INTRODUCTION

Bone is a dynamic tissue, tightly regulated by crosstalk between mesenchymal stem cell‐derived osteoblasts and haematopoietic‐derived osteoclasts. Intercellular signalling among cells and their progeny give rise to the canonical bone regulatory pathways that determine homeostatic balance in bone remodelling. Modification of these intercellular dynamics can result in anabolic or catabolic phenotypes. Beyond the role of osteoblasts and osteoclasts, mounting evidence support that macrophages can directly and indirectly contribute to bone anabolic and catabolic shifts.^1^, ^2^

The homeostatic cycle of bone remodelling is governed by both catabolic and anabolic phases working in a coordinated fashion. For example, during bone resorption, inflammatory cytokines including IL1, IL6 and TNFα are released, initiating the bone‐formative phases of the bone remodelling cycle.^3^ While the acute and highly regulated inflammatory chemokine/cytokine mediators remain essential stimulators of bone formation, aberrant release in amount, gradient or location can negatively impact physiologically balanced mechanisms, shifting homeostatic events towards disease.^4^ Inflammatory mediators regulate proliferation, migration and differentiation of mesenchymal stem cells (MSCs). The effects of IL1, IL6 and TNFα have been extensively studied in bone yet data pertaining to osteogenic differentiation remain controversial.^5^, ^6^ Croes et al. demonstrated that inflammatory factors enhance the osteogenic capacity of MSCs.^7^ In fracture healing context, CCR2‐mediated recruitment of inflammatory macrophages are indispensable for optimal endochondral ossification.^8^ Cytokines and chemokines (IL6 and CCL2) released from these inflammatory cells stimulate osteogenic differentiation of osteoprogenitor cells.^3^

An emerging area of interest in cell–cell signalling and biological crosstalk includes the discovery and understanding of ‘packaged cytokine/chemokine cargo’ called exosomes. Exosomes are extracellular nanosized bilayer phospholipid vesicles which mediate cell–cell communication in both physiological and pathological conditions carrying cargo consisting of a myriad of proteins, lipids, RNAs and microRNAs.^9^ They offer therapeutic advantages including biocompatibility, reduced immunogenicity and transportation of cargo contents without degradation due to the nature of the exosome bilipid capsule.^10^ Recent data demonstrate that exosomes derived from osteoblasts, MSCs, osteoclasts and macrophages have therapeutic potential in osteoporosis and regenerative medicine.^4^, ^11^, ^12^

Osteoblasts have been shown to communicate with other cell phenotypes through secretion of exosomes^13^ and exosome‐derived microRNAs exhibit a potential to direct osteogenesis and osteoclastogenesis.^14^, ^15^ However, there is a gap in the literature regarding the effects of preosteoblast‐derived exosomes on bone marrow macrophages and bone remodelling dynamics. As such, the purpose of this study was to explore the effects of preosteoblast‐derived exosomes on bone marrow macrophages and osteoblasts in vitro and in vivo.

## 2 MATERIALS AND METHODS

### 2.1 Isolation of macrophages

Bone marrow‐derived macrophages (BMMΦ) were isolated from 4‐ to 6‐week male C57BL/6J mice by flushing the femur and tibia with minimum essential medium eagle‐alpha (αMEM) supplemented with L‐glutamine, antibiotic 1× and 10% fetal bovine serum (FBS; Atlas Biologics, Fort Collins, CO) in the presence of macrophage colony stimulating factor (M‐CSF) (30 ng/mL, Peprotech).^16^ After 4 days in culture, significant of F4/80+ enriched macrophage population was observed by flow cytometry.^17^ Macrophages were plated independently at 2 × 10^6^ cells/well in 6‐well plates in αMEM.

### 2.2 Isolation of exosomes

MC3T3‐E1 clone 4 preosteoblastic cells (MC4, passage 11–13) were plated at 4 × 10^4^/cm^2^ in 150cm^2^ cell culture dishes with 10% FBS in αMEM. To optimize exosome isolation, different cell densities were evaluated. The next day (70%–80% confluency), media were changed with αMEM supplemented with 1% exo‐free FBS. After 24 h, conditioned media were collected, centrifuged and filtered with 0.22 μm Steriflips (MilliporeSigma) to eliminate cell debris. Exosomes were isolated according to the exosomes precipitation reagent (ExoQuick, System Biosciences) protocol. Exosomes pellets were dissolved in PBS, checked for mycoplasma and stored at −80°C until use.

### 2.3 Nanoparticle tracking analysis (NTA)

Size and number of particles were analysed using a NanoAnalyzer (NanoSight300, Malvern Panalytical) equipped with nanoparticle tracking NanoSight NS300 NTA software. Each exosome preparation was administered via syringe (five times) with video recording of Brownian motion recorded for 1 min. Exosome batches were checked with NTA prior to in vitro and in vivo use.

### 2.4 Transmission electron microscopy (TEM)

TEM was carried out using a Thermo Fisher Scientific Talos F200X operated at 200 kV. Exosomes in PBS (20 μL) were allowed to dry (~10 min) on carbon mesh at room temperature (RT). Images were acquired via bright‐field scanning TEM, with a collection angle of 0–10 mrad. Exosomes were checked by NTA and TEM to confirm particle size and shape before and after freezing (−80°C).

### 2.5 Western blot analysis

Western blot analysis was performed to analyse CD63 (exosome marker) expression. Cell Lysis Buffer (Cell Signaling Technology) was added to MC4 exosomes (MC4exo) in PBS, sonicated and total protein concentration measured via Bio‐Rad Protein Assay Dye Reagent Concentrate. Total protein (10 μg) was resolved by SDS‐PAGE and transferred to a PVDF membrane in a Mini Trans‐Blot Cell (Bio‐Rad Laboratories Inc.) at 100 V for 2 h in Novex BoltTM transfer buffer (Thermo Fisher Scientific). After blocking with 5% skim milk in Tris‐buffered saline with Tween‐20 (TBS‐T) at RT (1 hr), the PVDF membrane was incubated with anti‐CD63 rabbit antibody [EPR21151] (1:5000; Abcam) overnight at 4°C and probed with anti‐rabbit IgG, HRP‐linked antibody (1:2000; Cell Signaling Technology). Chemiluminescent detection was performed using SuperSignal™ West Femto Maximum Sensitivity Substrate (Thermo Fisher Scientific) in a ChemiDoc image system (Bio‐Rad Laboratories Inc.).

### 2.6 Exosome uptake

Preosteoblast‐derived exosomes were tagged with PKH67 (PKH67 Green Fluorescent Cell Linker kit, (Sigma). MC4exo were mixed with diluted PKH67 and incubated at 37°C for 5 min.^18^ Following incubation, exosome‐free medium containing FBS (10%) was added to stop the reaction. BMMΦ were seeded onto glass cover slips and placed in 6‐well plates. Tagged‐exosomes were added (1000 particle/cell). After 2, 24 and 48 h, cells were fixed with 4% paraformaldehyde (PFA) for 30 min and permeabilized with 0.5% Triton X‐100 in PBS for 10 min. Cells were then incubated with Phalloidin‐AlexaFluor660 (ThermoFisher) for 30 min and nuclei were stained with DAPI (ThermoFisher). Fluorescence was observed using the Leica THUNDER imaging system (Leica Microsystem). MC4exo uptake was confirmed via z‐stack imaging taken at 63x magnification.

### 2.7 Live and dead cell imaging

BMMΦs were plated into 96‐well plate (10^4^/well). Cells were treated with PBS (vehicle) or MC4exo in PBS (10^3^ particles/cell) every other day. Cell viability was assessed on day (d) 2 and 7 using the Live/Dead Cell Imaging Kit (ThermoFisher Scientific) and imaged using Leica THUNDER.

### 2.8 Quantitative polymerase chain reaction (qPCR)

BMMΦs from 3 separate mice were plated overnight at 2 × 10^5^/cm^2^. Cells were treated every other day with PBS with or without MC4exo (10^3^ particles/cell) in αMEM supplemented with 1% exo‐depleted FBS. Experiments were repeated three independent times.

Total RNA was isolated using the RNeasy® Mini Kit (#74104, Qiagen) at 20 h and d7. qPCR was performed using TaqMan gene expression master mix (#4369016, AppliedBiosystems) and TaqMan probes as follows: *Cd86* (Mm00444541_m1), *CD206* (Mm01329362_m1), *Il1β* (Mm00434228_m1), *Il10* (Mm01288386_m1), *Tnfα* (Mm00443258_m1), *Rankl* (Mm01288386_m1), *Ccl2* (Mm00441242_m1), *Nos* (Mm00440502_m1) and *18s* (Mm03928990_g1). Real time PCR was analysed on ABI PRISM 7700 (Applied Biosystems). Relative expression levels were calculated after normalization to *18s* expression.

### 2.9 Osteoclastic differentiation of macrophages

BMMΦ (*n* = 3) were plated into 6‐well plates (2 × 10^5^/cm^2^) in αMEM with 1% exo‐depleted FBS and M‐CSF (30 ng/mL). Groups included: untreated control group, MC4exo (10^3^ particles/cell), RANKL (100 ng/mL; Peprotech) and RANKL+MC4exo. Cells were treated every other day for 7d followed by tartrate‐resistant acid phosphatase (TRAP) staining. Positive cells with ≥3 nuclei were considered osteoclasts.

### 2.10 The effects of MC4exo on mRNA and mineralization of calvarial osteoblasts

Calvarial osteoblasts (cOB) were isolated from 7d‐old C57BL/6 mice calvaria. cOB (*n* = 3) were plated in 6‐well plates (5 × 10^4^/cm^2^) for RNA isolation and 12‐well plate for von Kossa staining. Cells were treated with or without MC4exo (10^3^ particle/cell) using αMEM with 1% exo‐depleted FBS every 2d. RNAs were isolated after 5d, 10d, 15d and 20d. Mineralized tissue‐associated genes including *Alp* (Mm00475834_m1), *Bglap* (Mm03413826_mH), *Col1a1* (Mm00801666_g1), *Runx2* (Mm00501584_m1), *Opg* (Mm00441906_m1), *Rankl* (Mm00441906_m1) and 18S (Mm03928990_g1) mRNA expressions were analysed by qRT‐PCR as described above. Von Kossa staining was performed after 21d to evaluate in vitro mineralization.

### 2.11 In vivo experiments

Mice were maintained in accordance with institutional animal care and use guidelines, and experimental protocols were approved by the Institutional Animal Care and Use Committee of the University of Michigan. Sample size was calculated a priori, using our previous intratibial study, estimating the amount of mice needed using a power analysis (*p* < 0.05, power = 0.8, effect estimate = 17–20%diff) calculation resulting in an approximate sample size of *n* = 10 to obtain significant and meaningful data. We used 5 more/gp to control for exclusion possibilities of failed injections, variability outliers (data 2 standard deviations from the mean) or animal adverse consequences. Thirty 8‐week‐old male C57Bl/6J mice (Jackson Labs, Bar Harbor, ME) were randomized into groups as vehicle (20 μL PBS, *n* = 15) and MC4exo in PBS, 10^8^ particles/μL, 20 μL, *n* = 15). Mice were anaesthetized by isoflurane and the knee was denuded and sterilized with provo‐iodine. A 25G needle was inserted through the cortex of the anterior tuberosity of the tibia in a rotating drill‐like movement to minimize cortical fracture and to create a hollow core ~3 mm into the diaphysis. Another syringe (29G needle) prefilled with either vehicle or exosomes (20 ul) was reinserted into the core and injected while the syringe was slowly withdrawn to allow the bone core to fill. Injections were performed with no knowledge of substance injected. Six mice were discarded due to unsuccessful inoculation (the only exclusion implemented) leaving 24 mice (12 vehicle, 12 MC4exo) for analysis. Animals were given analgesic for 24 h post‐injection. Spleen, serum and tibia were collected at 4 weeks post‐injection. These methods are in compliance with the ARRIVE guidelines.

### 2.12 Micro‐computed tomography

Tibiae were fixed in 4% PFA for 72 hr at 4°C, transferred to 70% EtOH, then analysed via micro‐computed tomography (μCT; Scanco Medical) at 12 μm voxel size as previously described.^19^, ^20^ μCT analyses were performed blindly twice by two independent researchers. ‘Whole’ tibia trabecular bone was examined from the top of the growth plate to 7.2 mm distally. Metaphyseal trabecular bones were measured from the top of the growth plate to 1.8 mm distally and diaphyseal trabecular bone was examined from this point to 5.4 mm distally. 0.36 mm of cortical bone was analysed starting at 2.64 mm above the fibula–tibial junction.

### 2.13 Immunofluorescence and histological staining

Following μCT, bones were decalcified in 14% ethylenediaminetetraacetic acid (EDTA; Sigma) for 2 weeks. Staining was performed on deparaffinized and rehydrated 5 μm sections. Antigen was retrieved via 50 μg/mL proteinase K for osteocalcin (Ocn) and Collagen type 1 (Col1a1) staining, and using 0.1% trypsin for F4/80 and osterix (Osx) staining. Non‐specific binding was blocked with 10% normal goat serum/FBS (1 hr). Sections were incubated with unconjugated primary antibodies against Col1a1 (United States Biological), F4/80 (Abcam), Ocn (Thermo Fisher Scientific) or Osx (Abcam). The species‐matched secondary antibody used was goat‐anti‐rat‐AlexaFluor647 or goat‐anti‐rabbit‐AlexaFluor680. Slides were mounted using Prolong Gold mounting medium with DAPI (Thermo Fisher Scientific). Haematoxylin and eosin staining was performed using standard protocols. TRAP was performed as previously described.^21^ Slides were visualized using the Leica THUNDER.

### 2.14 Histomorphometry

All staining were manually evaluated using ImageJ. For TRAP, Ocn and F4/80 staining, the length of positive staining per bone surface was measured. For Osx, the number of positive signals per bone surface was quantified.

### 2.15 ELISA

Blood was allowed to coagulate at RT for 30 min then centrifuged (20 min; 8000 rpm). Serum was collected and stored at −80°C until assay. Serum markers of bone resorption (TRAcP5b) and bone formation (P1NP) were measured using ImmunoAssay analytics (ImmunoDiagnosticSystems). CCL2 was quantitatively measured using the RayBio® Mouse enzyme‐linked immunosorbent assay (ELISA) assay system (RayBiotech, Inc).

### 2.16 Statistics

Statistical analyses were performed using GraphPad Prism (GraphPad Software, version 9.5.0). Parametric tests were performed on data sets that passed Shapiro–Wilk normality test using two‐tailed unpaired *t*‐test, two‐way anova with Sidak's multiple comparisons test or one‐way anova with Tukey's post hoc test. When sample size was too small for the Shapiro–Wilk test, normal distribution was assumed. A *p*‐value of <0.05 was considered statistically significant. Data are presented as mean ± SEM.

## 3 RESULTS

### 3.1 Characterization of preosteoblast‐derived exosomes

**FIGURE 1:**
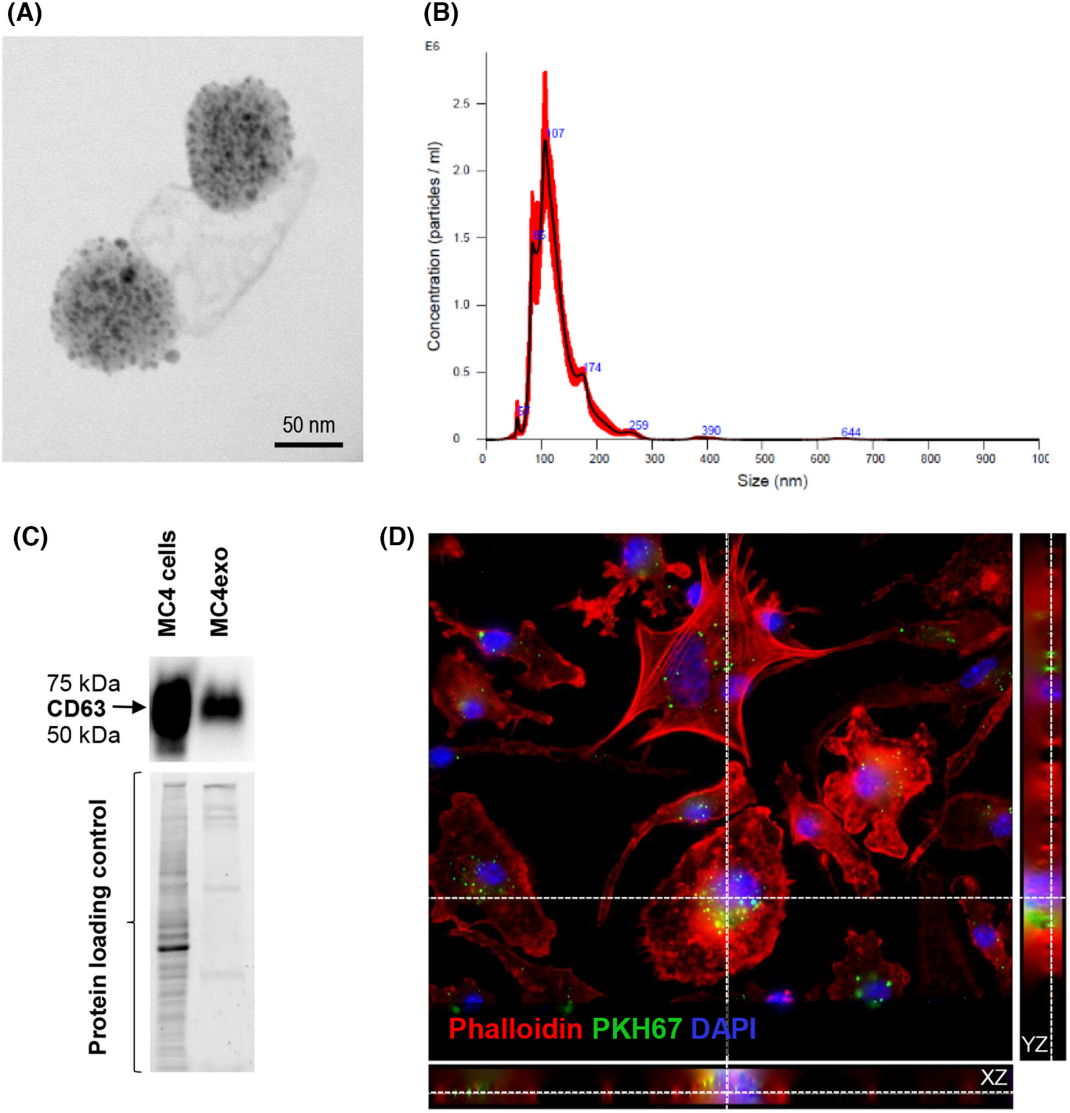
Characterization of the exosomes isolated from MC3T3‐E1 #clone 4 (MC4) cells. (A) Transmission electron microscopy images of exosomes isolated from MC3T3‐E1 cells. (B) Particle number and size of MC4 exosomes (MC4exo) as determined by Nanosight 300. (C) Western blot analysis for CD63 (exosome marker) expression in MC4 cells and isolated MC4exo including protein loading controls. (D) Z stack images of bone marrow‐derived macrophages cultured with PKH67‐labelled MC4exo showing intracellular localization. Original magnification: 63x. Red ‐ Phalloidin (Actin), green ‐ PKH67 (exosomes), blue ‐ DAPI (nucleus). Results are representative of at least three independent experiments.

TEM images demonstrated that MC4exo were uniform in shape and size (Figure 1A). NTA analysis revealed that the average particle size was 126.5 ± 3.7 nm, the proportion of particles with a size ranging from 87.3 ± 3.8–179.3 ± 5.5 nm was 90% (Figure 1B) and the particle density was 1.34 × 10^11^ particles/ml. Western blotting confirmed CD63 expression in both MC4 cells and isolated MC4exo (Figure 1C).

### 3.2 Effects of exosomes on BMMФ viability, gene expression and osteoclastic differentiation

To examine whether preosteoblast‐derived exosomes were taken up by macrophages, BMMФ were co‐cultured with MC4exo pre‐labelled with PKH67. Z‐stack imaging confirmed intracellular localization of PHK67^+^ MC4exo, reflecting engulfment by macrophages (Figure 1D). Further examination of BMMФ cultured with MC4exo implied that MC4exo treatment affected morphology at 3 and 7 days (Figure S1A), but had no effect on cell viability (Figure S1B).

**FIGURE 2:**
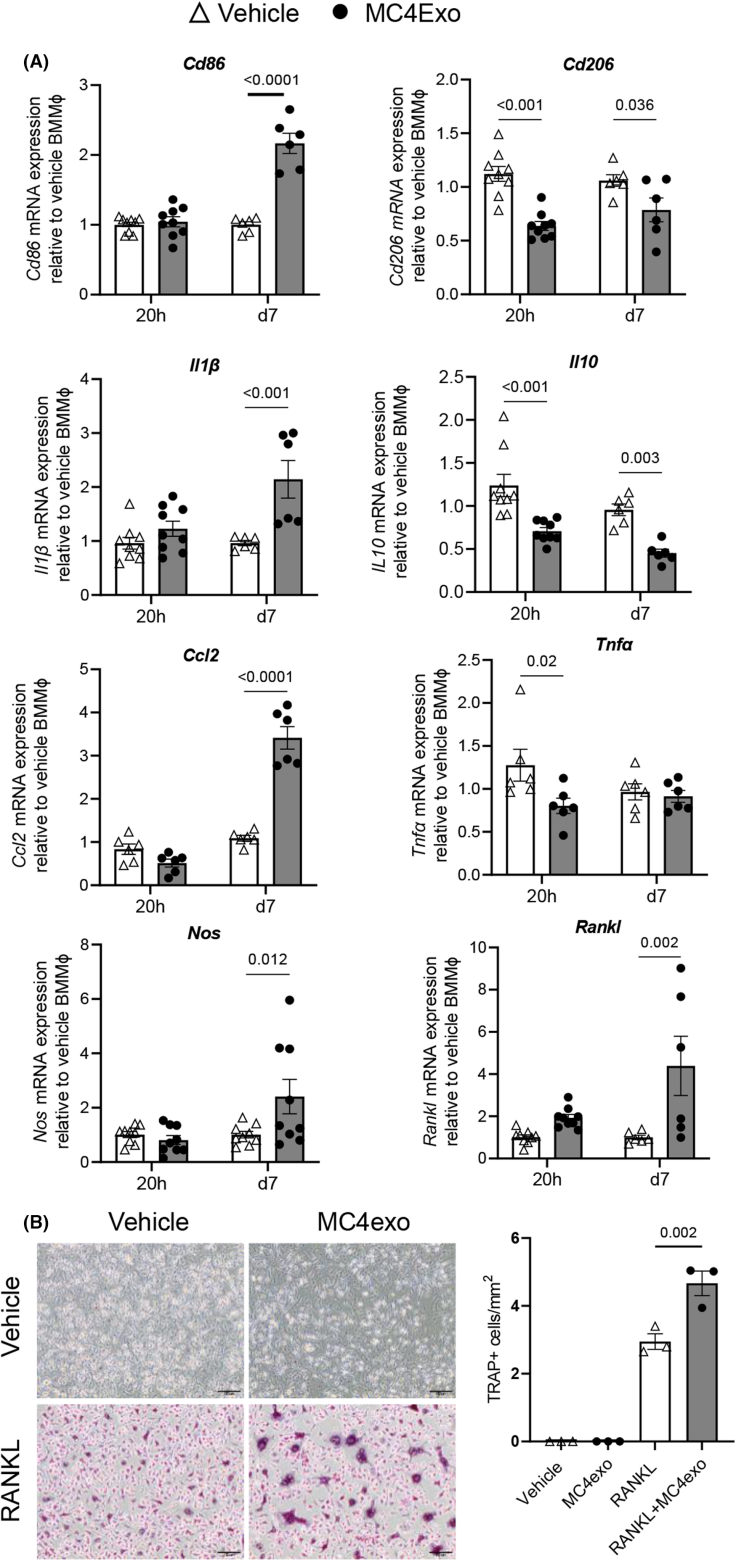
BMMΦs cytokine expression and osteoclastogenic differentiation in response to MC4exo exposure. (A) *Cd86, Cd206, Il1β, Il10, Ccl2, Tnfα, Nos and Rankl* mRNA expressions of BMMΦ cultured with MC4exo at 20 h and day 7 post exposure. (B) TRAP staining of BMMΦs cultured in the presence of vehicle or RANKL plus vehicle of MC4exo and quantification of the number of TRAP+ osteoclasts (≥3 nuclei).

Assessment of BMMФ gene expression profiles via qPCR at 20 h showed that MC4exo exposure reduced *Cd206* and *Il‐10* and this reduction was sustained until day 7. *Tnfα* was reduced at 20 h but not at day 7. Increased *Cd86*, *Il1β, Ccl2 and Nos* were detected at day 7 but not at 20 h. Notably, MC4exo exposure increased BMMФ *Rankl* (Figure 2A). This increase did not translate in augmented RANKL in the media at 24 h or day 7 (Figure S1C). The osteoclastic differentiation potential of BMMФs was next assessed. In the absence of RANKL, BMMФ cultured with MC4exo or vehicle did not differentiate into osteoclasts (Figure 2B). While TRAP^+^ osteoclasts (≥3 nuclei) were detected in both RANKL‐treated groups, MC4exo exposure enhanced osteoclastic differentiation compared with the vehicle‐treated group (Figure 2B).

### 3.3 Effects of preosteoblast‐derived exosomes on calvarial osteoblasts (cOBs)

**FIGURE 3:**
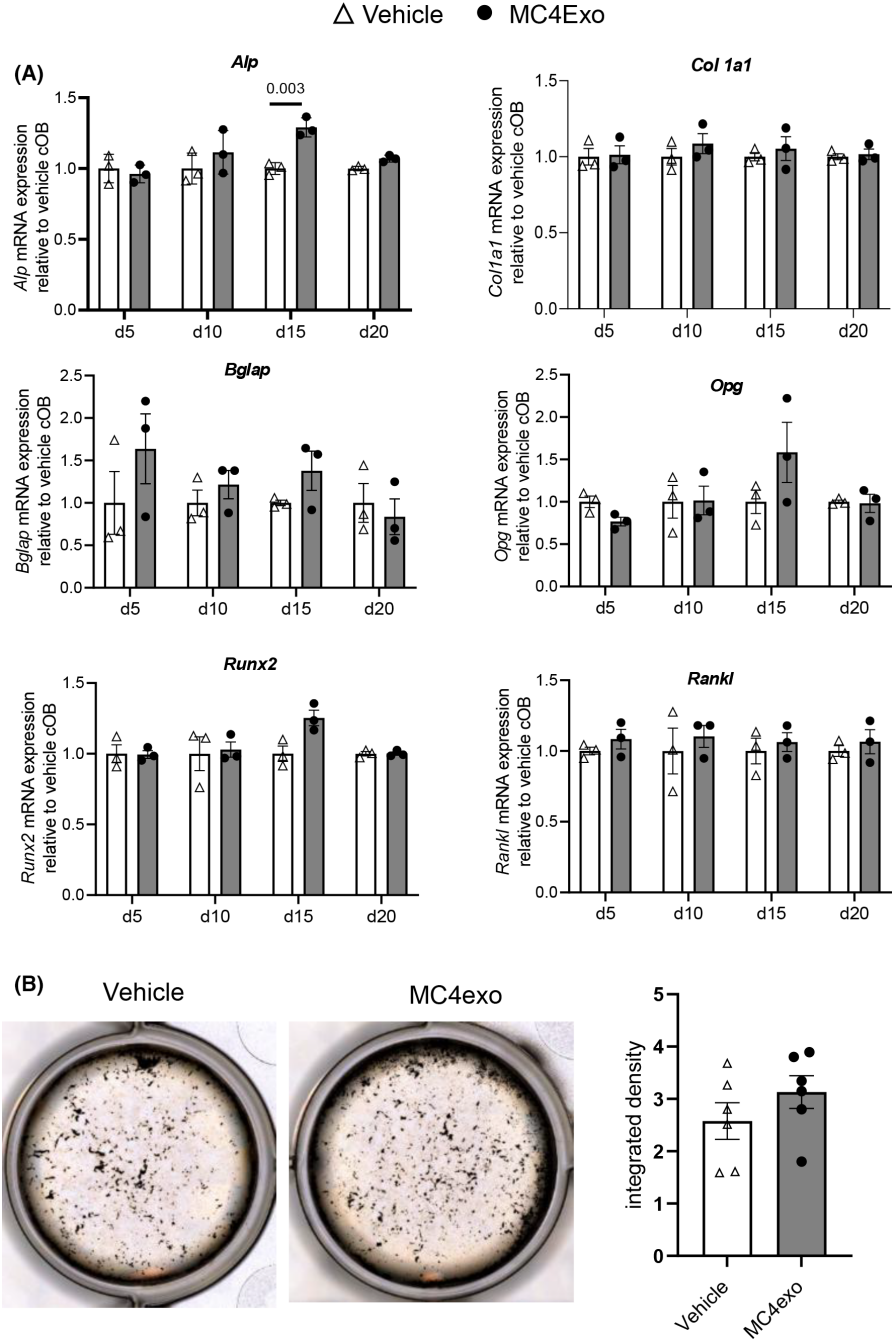
MC4exo effects on the osteogenic capacity of calvarial osteoblasts. (A) The effects of MC4exo on the mineralized tissue associated gene expression of calvarial osteoblasts on days 5, 10, 15 and 20: Collagen type I (*Col1a1*), Bone GLA protein (*Bglap*), Alkaline phosphatase (*Alp*), Osteoprotegerin (*Opg*), *Runx2* and *Rankl* mRNA expressions. (B) Von Kossa staining of calvarial osteoblasts treated with MC4exo or vehicle every 2d for 21d. Image J was used to analyse mineralized nodules. Results are from two independent experiments. *n* = 6/group.

Gene expression profiles of cOBs exposed to either MC4exo or vehicle were examined by qPCR at days 5, 10, 15 and 20 post treatment. No significant difference was observed with *Col1a1*, *Bglap*, *Opg* and *Rankl* at any of the time points assessed while an increase in *Runx2* was detected only at d15. *Alp* was also increased at d15 and this was sustained until d20 (Figure 3A). MC4exo treatment had no impact on cOBs mineralization (Figure 3B).

### 3.4 MC4exo increased fractional bone volume and bone mineral density

**FIGURE 4:**
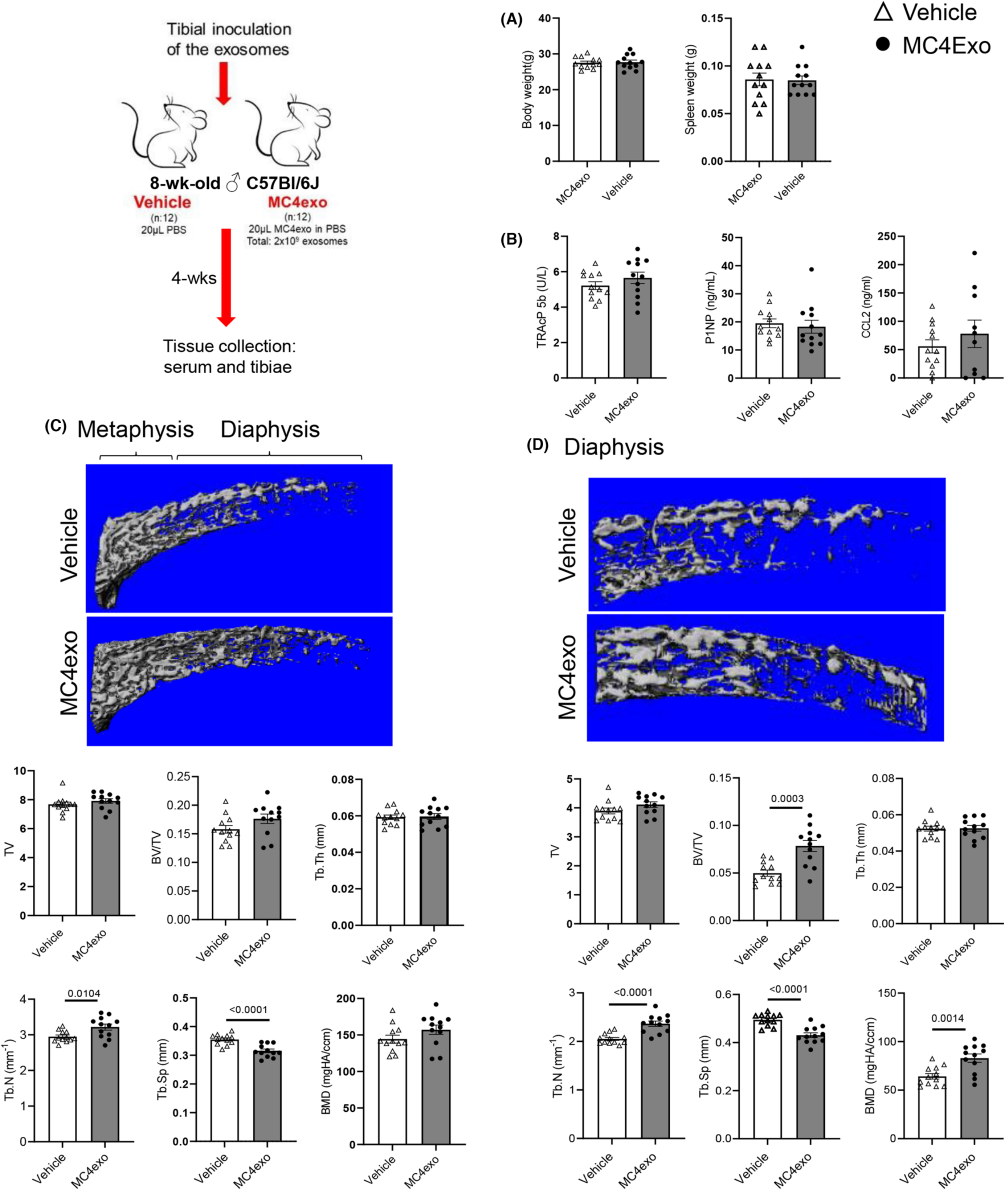
Intratibial injection of MC4exo increased trabecular bone in the diaphysis. (A) Body weight and spleen weight at experimental endpoint. (B) Serum levels of TRAP‐5b, P1NP and CCL2. μCT analysis of the trabecular bone at 4 wks after intratibial injection with MC4exo or vehicle in the entire tibia (C) or diaphyseal region (D): tissue volume (TV), bone volume per total volume (BV/TV), trabecular thickness (Tb.Th), trabecular number (Tb.N), trabecular spacing (Tb.Sp) and bone mineral density (BMD). *n* = 12/group.

To examine the impact of preosteoblast‐derived exosomes on bone and bone turnover markers in vivo, MC4exo or PBS (vehicle) were injected intratibially. The treatment had no impact on total body weight or spleen weight (Figure 4A) or serum levels of TRAcP5b and P1NP (Figure 4B). As *Ccl2* gene transcripts were elevated in BMMФ exposed to MC4exo, we also examined serum CCL2 but found no difference between groups (Figure 4B). μCT evaluation of the trabecular bone within the entire injected tibiae showed that while there was no difference in fractional bone volume, there was a significant increase in trabecular number and reduction in spacing (Figure 4C). When analyses were compartmentalized to metaphyseal versus diaphyseal regions, we found marked increase in trabecular BMD and fractional bone volume in the diaphyses of MC4Exo‐treated mice due to increased number and reduced trabecular spacing (Figure 4D). MC4exo inoculation had no impact on any cortical bone parameters examined (Figure S2).

**FIGURE 5:**
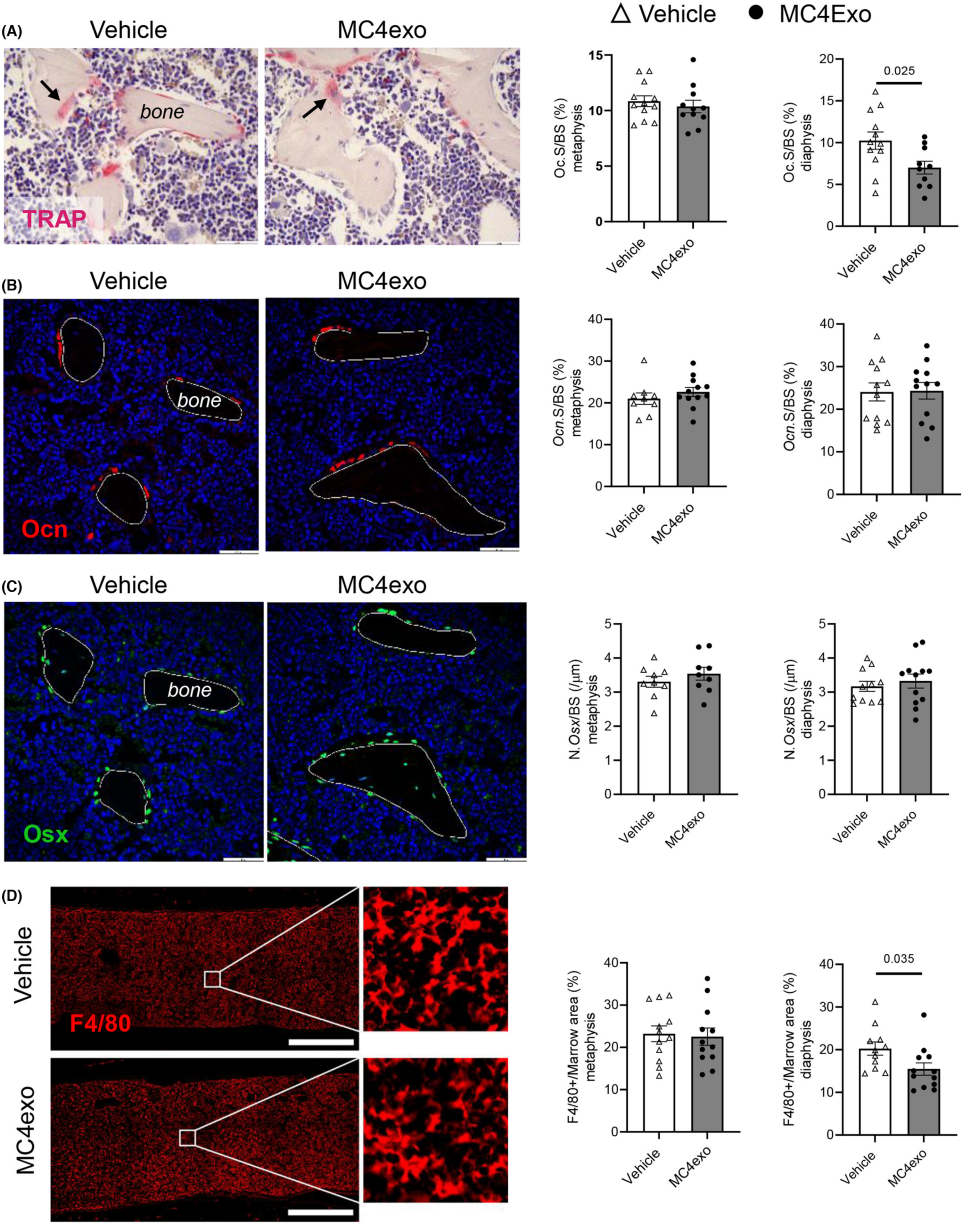
Intratibial injection of MC4exo had no impact on osteoblast frequency but reduced osteoclasts and macrophages at the experimental end‐point. Histological and immunofluorescent analysis of the tibia. (A) TRAP staining in the diaphysis and quantification of was performed on tibia paraffin sections and analysis included. Representative TRAP^+^ surface per trabecular bone surface in the metaphysis and diaphysis stained images. Black arrows indicate TRAP^+^ osteoclasts. Reduced TRAP staining was observed in the diaphysis of the tibia. (B) Immunofluorescent staining for osteocalcin (Ocn) (red) was performed. *n* = 12 (Vehicle); *n* = 12 (MC4exo). Scale bar; 500 μm. No significant difference was noted. (C) Immunofluorescent staining for osterix (green) was performed. *n* = 12 (Vehicle); *n* = 12 (MC4exo). No significant difference was found. (D) Immunofluorescent staining for F4/80 was performed. Significant reduction was found in the diaphysis of the tibia. ∆: Vehicle, ●: MC4exo. Scale bar: 500 μm.

Increased mineralized tissue was apparent in Col1a1 staining of tibial sections (Figure S3A). Bone cell markers were next examined in tissue sections. Given the differential impact of MC4exo injection on metaphyseal and diaphyseal trabecular bone, each region was examined separately as per Figure S3B. There was a reduction in TRAP^+^ osteoclasts in the diaphysis but not metaphysis (Figure 5A) while osteoblast markers Ocn (Figure 5B) and Osx (Figure 5C) were unaltered in both compartments. Assessment of F4/80 showed significant reduction only in the diaphysis (Figure 5D).

## 4 DISCUSSION

The role of macrophage polarization and their protein release profiles in the context of bone formation and healing remains poorly understood. There is value in assessing modulation of inflammatory signals as strategies to direct bone homeostasis in cases of hard tissue disease profiles and biased resorptive phenotypes including osteoporosis, bone trauma, infection, osteonecrosis and periodontal diseases.^4^ Polarization of macrophages and differentiation to osteoclasts depends heavily on cell–cell crosstalk, cytokine signalling, location/cytokine gradients and microenvironment‐specific proteins.^22^ Macrophages have been identified as regulators of destructive inflammation in pathologic situations like arthritis; however, recent evidence suggests that in bone homeostasis macrophages can support bone mass accrual and leverage bone repair.

Despite the emerging roles of macrophages in bone homeostasis and pathology, there is limited data discerning the effects of macrophage polarization on osteoclastogenesis. Assessment of the regulatory effects of macrophage polarization (M0, M1 and M2) on RANKL‐induced osteoclastogenesis showed that M1 macrophages suppress osteoclastogenesis while M0 or M2 macrophages had no regulatory effects.^23^ In the present study, preosteoblast‐derived exosomes stimulated a proinflammatory cytokine response in macrophages, signifying an M1‐like phenotype. Our study demonstrated that co‐treatment with MC4exo enhanced RANKL‐induced osteoclastogenesis in BMMФs. Although MC4exo increased RANKL mRNA expression in BMMФs, this alone did not alter the amount of soluble RANKL. While membrane‐bound RANKL expression was not examined herein, our findings demonstrated that any MC4exo‐induced changes in BMMФs were insufficient to trigger osteoclast formation under the experimental conditions examined. Osteoclasts are required for the invasion of blood vessels at the initial step of bone marrow cavity formation.^24^ Their conclusion might explain the increase in trabeculae in the present study in correlation with the enhanced osteoclastic differentiation induced by MC4exo. Preosteoblast‐derived exosomes increased bone mineral density, bone volume and trabecular number in vivo. We may speculate that increased osteoclastic differentiation may hasten bone marrow cavity formation and the turnover of bone remodelling. TRAP staining in the diaphysis of the tibia was reduced with MC4exo at 4 weeks. It is possible that osteoclastic activity was modulated during early resolution and reduced at a later healing phase. Assessment of osteoclastogenesis at an early post‐injection time point would clarify this and would also inform whether MC4exo‐induced cellular changes are time‐dependent.

Many studies demonstrated that bone MSCs (BMSCs)‐or osteoblast‐derived exosomes induced M2‐like phenotype in macrophages and M2‐like macrophages stimulated bone formation.^25^, ^26^, ^27^, ^28^, ^29^ In contrast to these studies, we found that osteoblast‐derived exosomes induced an M1‐like phenotype inducing CD86 and decreasing CD206 (MRC1). However, we observed increased bone mineral density, and fractional bone volume in vivo. These findings suggest that macrophage proinflammatory responses may shorten the inflammation period and accelerate the initial phase of resolution. Acute induction of inflammation and osteoclast differentiation by preosteoblast‐derived exosomes may hasten an enhanced bone formation.

You et al. reported that BMSCs‐derived exosomes enhance proliferation, osteoblastic differentiation and ALP activity of human osteoblasts (hFOB1.19).^30^ These authors reported that BMSC‐derived exosomes contain miR‐21‐5p, which can regulate hFOB1.19 cell activities through Kruppel‐like factor 3 (KLF3) targets. Additionally, Wang et al. reported that osteoblast‐derived exosomes containing miR‐503‐3p inhibit differentiation of osteoclast progenitors by downregulation of heparanase (Hpse).^14^ In the present study we did not elucidate the cargo of the exosomes.

The effects of extracellular vesicles of mouse osteoblasts or BMSC on osteoclasts have been investigated.^22^, ^31^ Some studies reported osteoclast‐derived vesicles decrease osteogenesis of osteoblasts.^32^, ^33^ while Liang et al.^34^ demonstrated that osteoclast‐derived extracellular vesicles increased osteogenesis of bone marrow stem cells. According to the origin of the cells and source of the extracellular vesicles, the findings of the studies show differences. In the present study, we checked the effects of MC4exo on the mouse BMMФs and primary calvarial osteoblasts to clarify which cells primarily dominate the regulation of bone formation. We observed that while preosteoblast‐derived exosomes induced a proinflammatory response in BMMФ, they had no significant effects on calvarial osteoblast mineralization and mineralized tissue‐associated genes. These findings suggest that the effect of preosteoblast‐derived exosomes in bone is mediated by macrophages. A recent paper by Uenaka et al. reported that osteoblast‐derived vesicles induce a switch from bone‐formation to bone‐resorption in vivo.^15^ They found osteoclastic activity in both in vitro and in vivo. In the present study, we did not observe induced osteoclastic activity in the in vivo experiments. There are some major differences in the methodology in our study versus Uenaka's study.^15^ They used matured primary osteoblasts or MC3T3‐E1 cells and maintained the cells in the osteogenic medium before vesicle isolation whereas we used preosteoblastic MC3T3‐E1 cells without any osteogenic induction for exosomes isolation. Additionally, they evaluated osteoblast‐derived vesicles on the calvarial bones while we used tibia model. The dynamic of the calvarial bones and the spongious/cortical bone ratio is totally different from the tibia. The tibia injection model provides good model to evaluate both the cancellous and compact bone. We used nanosized vesicles while they described their vesicles as small osteoblast vesicles. Furthermore, the endpoint of the animal experiments of Uenaka was 8 weeks for cranial bone whilst we performed histological and radiological evaluations at 4 weeks. Osteoclastic activity may show different time rhythm during bone remodelling for short and long terms. In the present study we used preosteoblastic mouse MC3T3‐E1 Clone 4 cells for exosome isolation to provide better standardization in cargo content of exosomes. However, the extracellular vesicle field may generally suffer from a lack of reproducibility. There is no universal agreement on many aspects of methodology in extracellular vesicle research, including the best methodology for enrichment, and protocols vary between laboratories. These technical challenges may complicate the interpretation of the results.^35^

In conclusion, a single local inoculation of the tibia with preosteoblast‐derived exosomes significantly changed bone architecture, increasing trabeculae and bone mineral density. These findings suggest that preosteoblast‐derived exosomes can be considered for treatment of bone diseases, where their action targets macrophages, regulates osteoclast differentiation and the activity of cells in bone which is critical for angiogenesis and bone formation.^19^ Preosteoblast‐derived exosome‐based treatments are promising candidates for osteoporosis, osteonecrosis, bone fracture and regenerative medicine, and dentistry.

## AUTHOR CONTRIBUTIONS

**Sema S. Hakki:** Conceptualization (lead); data curation (lead); formal analysis (lead); investigation (lead); methodology (lead); validation (lead); writing – original draft (lead); writing – review and editing (equal). **Lena Batoon:** Data curation (equal); formal analysis (equal); investigation (equal); methodology (equal); writing – review and editing (equal). **Amy J Koh:** Conceptualization (equal); funding acquisition (equal); methodology (equal); validation (equal); writing – review and editing (equal). **Rahasudha Kannan:** Investigation (equal); methodology (equal); writing – review and editing (equal). **Veronica Mendoza‐Reinoso:** Methodology (equal); project administration (equal); writing – review and editing (equal). **John Rubin:** Methodology (equal); supervision (equal); writing – original draft (equal); writing – review and editing (equal). **Laurie K McCauley:** Conceptualization (lead); project administration (lead); resources (equal); supervision (equal); writing – review and editing (equal). **Hernan Roca:** Conceptualization (equal); funding acquisition (lead); investigation (equal); methodology (equal); project administration (lead); supervision (equal); writing – review and editing (equal).

## CONFLICT OF INTEREST STATEMENT

The authors confirm that there are no conflicts of interest.

## Supporting information


Figure S1–S3.

Data S1.

## Data Availability

The data that support the findings of this study are available from the corresponding author upon reasonable request.
