# Supplementary figures and images for: The effects of preosteoblast‐derived exosomes on macrophages and bone in mice
Source: J Cell Mol Med. 2023 Nov 6;28(1):e18029. doi: 10.1111/jcmm.18029 (PMC10805488; doi:10.1111/jcmm.18029)

Supplemental Figure 1

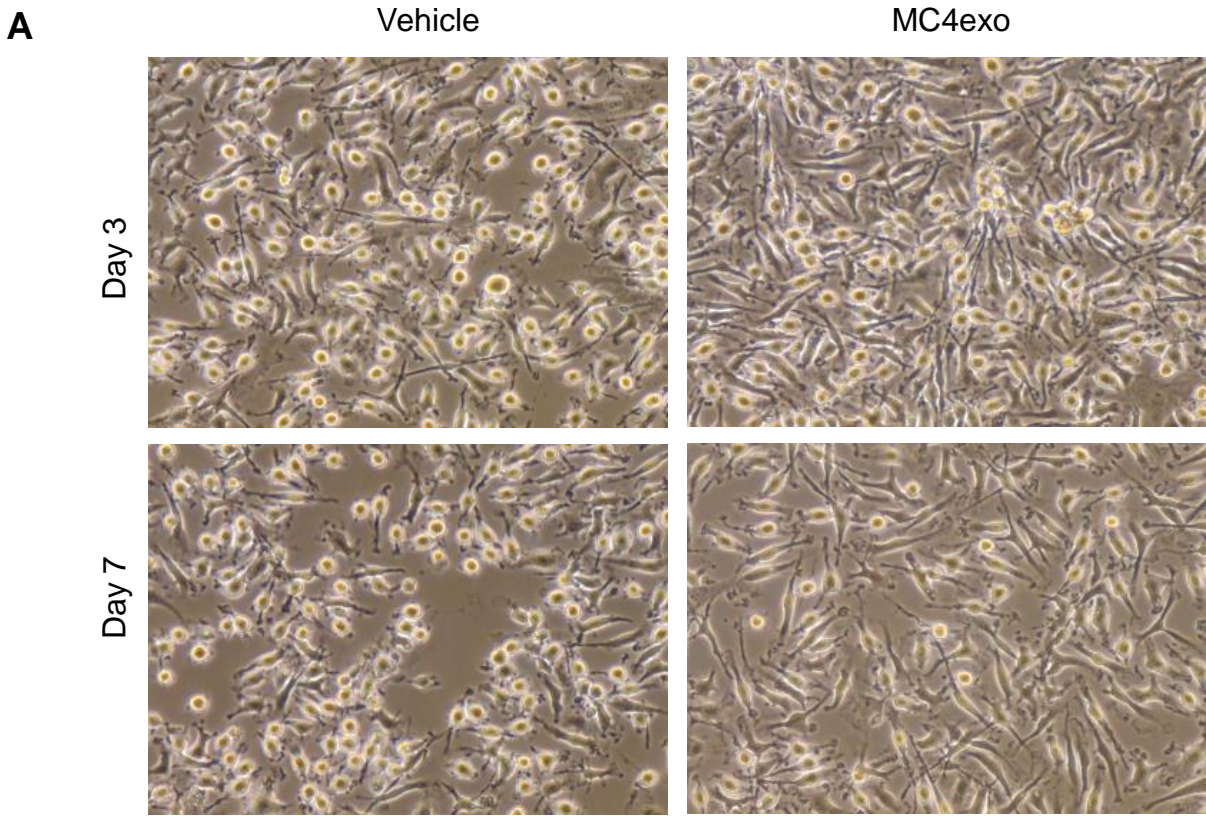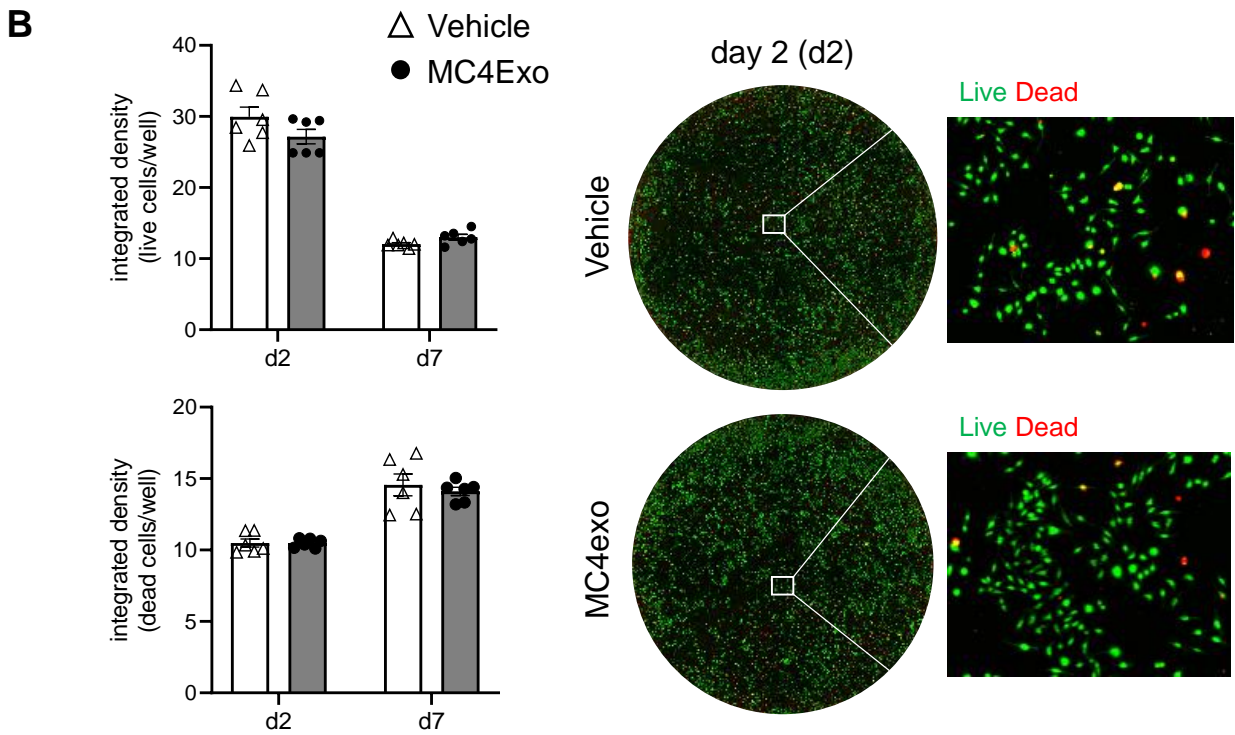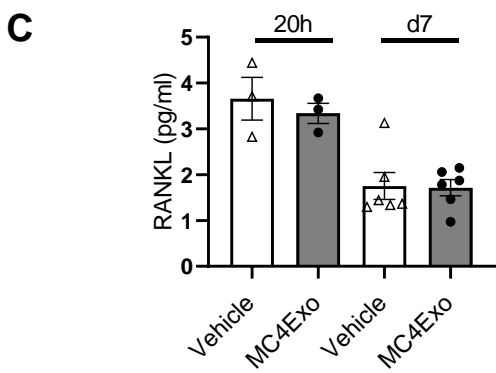

Supplemental Figure 2

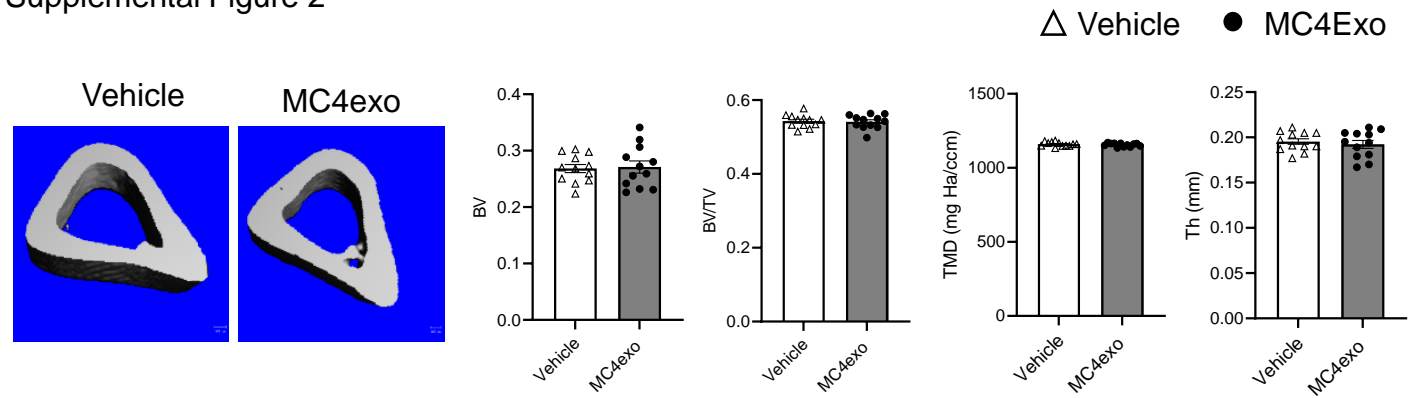

Supplemental Figure 3

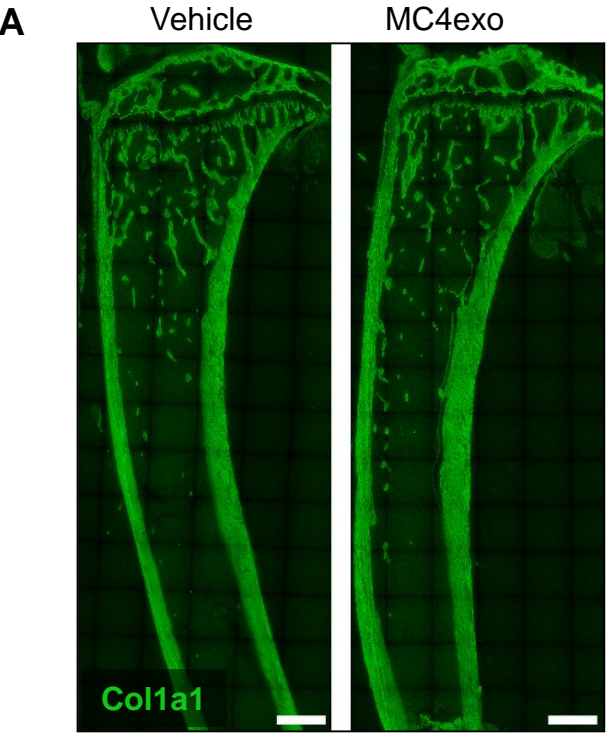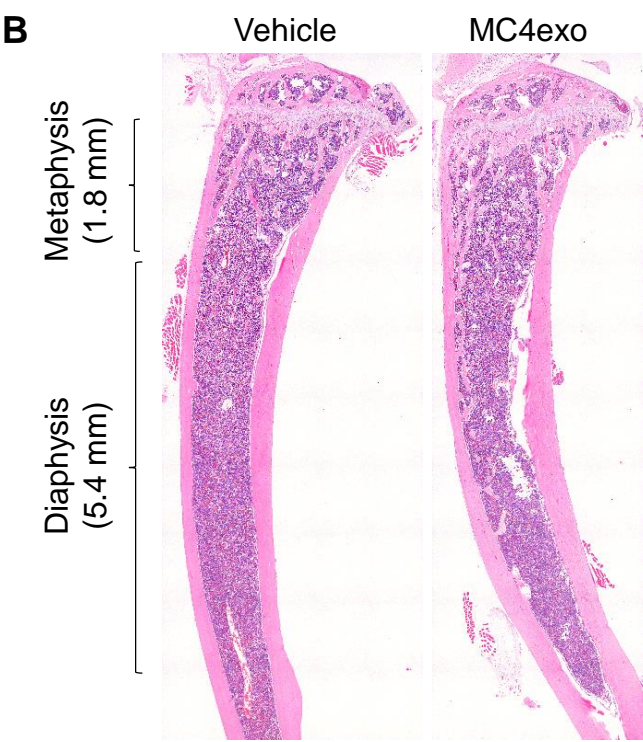

Supplement: Supplementary file 1 — Figure S1–S3. [file JCMM-28-e18029-s002.pdf]
