# Supplementary material for: The effects of preosteoblast‐derived exosomes on macrophages and bone in mice
Source: J Cell Mol Med. 2023 Nov 6;28(1):e18029. doi: 10.1111/jcmm.18029 (PMC10805488; doi:10.1111/jcmm.18029)
Supplement: Supplementary file 2 — Data S1. [file JCMM-28-e18029-s001.doc]

**Supplemental Figure Legends**

**Supplemental Figure 1.** A) The effects of MC4exo on BMMФ morphology and viability. Enhanced cellularity at day 3 and. A more spindle-shaped shape morphology was noted at day 7 in the cells treated with MC4exo. B) Live and dead cell staining of the BMMФ cultured with vehicle or MC4exo (10^3^ particles/cell) treatment. Green represents live cells while red represents dead cells. There was no significant difference in the survival of the macrophages on days 2 and 7. Graphical results are representative of two independent experiments. ∆: Vehicle, ●: MC4exo. C) Levels of RANKL protein in the media of BMMФ cultured with vehicle or MC4exo. Media were collected at 20h or day 7 post exposure.

**Supplemental Figure 2.** Intratibial injection of MC4exo had no impact on or cortical bone at 4wks post treatment: bone volume (BV), bone volume per total volume (BV/TV), tissue mineral density (TMD) and thickness (Th). n=12/group.

**Supplemental Figure 3**. Representative image of the metaphysis and the diaphysis of the tibia is shown. Representative images showing collagen type 1 (Col1a1)(A) and and H&E staining(B) of tibial sections from mice. Staining of the tibiae confirms more collagen matrix and trabeculation in the diaphysis in M.C4Exo. Bones were collected at 4wks. MC4exo treatment. Scale bar: 500µm (A). n=12 (vehicle); n=12 (MC4exo).
